# Demonstration and Analysis of the Suction Effect for Pumping Lymph from Tissue Beds at Subatmospheric Pressure

**DOI:** 10.1038/s41598-017-11599-x

**Published:** 2017-09-21

**Authors:** Samira Jamalian, Mohammad Jafarnejad, Scott D. Zawieja, Christopher D. Bertram, Anatoliy A. Gashev, David C. Zawieja, Michael J. Davis, James E. Moore

**Affiliations:** 1 Department of Bioengineering, Imperial College London, South Kensington Campus, London, SW7 2AZ United Kingdom; 2 Department of Medical Physiology, Texas A&M University Health Science Center College of Medicine, Temple, TX 77843 USA; 3 Department of Pharmacology and Physiology, University of Missouri School of Medicine, Columbia, MO 65212 USA; 4 School of Mathematics and Statistics, University of Sydney, Sydney, New South Wales 2006 Australia

## Abstract

Many tissues exhibit subatmospheric interstitial pressures under normal physiologic conditions. The mechanisms by which the lymphatic system extracts fluid from these tissues against the overall pressure gradient are unknown. We address this important physiologic issue by combining experimental measurements of contractile function and pressure generation with a previously validated mathematical model. We provide definitive evidence for the existence of ‘suction pressure’ in collecting lymphatic vessels, which manifests as a transient drop in pressure downstream of the inlet valve following contraction. This suction opens the inlet valve and is required for filling in the presence of low upstream pressure. Positive transmural pressure is required for this suction, providing the energy required to reopen the vessel. Alternatively, external vessel tethering can serve the same purpose when the transmural pressure is negative. Suction is transmitted upstream, allowing fluid to be drawn in through initial lymphatics. Because suction plays a major role in fluid entry to the lymphatics and is affected by interstitial pressure, our results introduce the phenomenon as another important factor to consider in the study of lymphoedema and its treatment.

## Introduction

**Figure 1:**
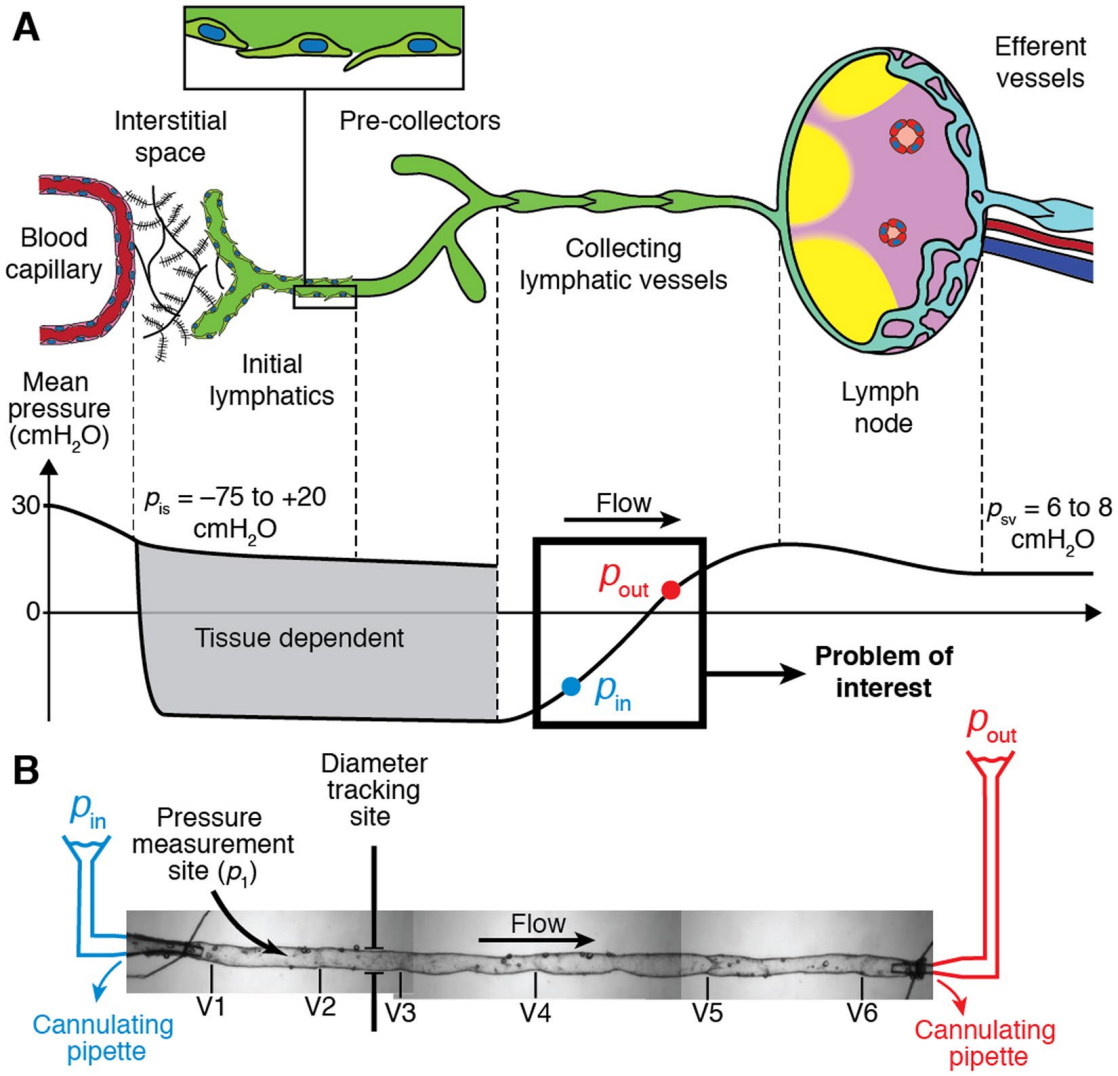
Lymph flow pathway. (**A**) Lymph flow pathway from formation in the interstitial space to return to the subclavian vein (Top) and indicative pressure values at each compartment (Bottom). Subatmospheric pressures have been measured in various interstitial spaces, initial lymphatics and pre-collectors. Previously, it was not known how the lymphatic system pulls in fluid from these spaces and delivers it, against the prevailing hydrostatic pressure gradient, into collecting lymphatics in which luminal pressure is positive, given that these vessels are only capable of contraction (“Problem of interest” as indicated). (**B**) An isolated, cannulated, and pressurized lymphatic vessel from rat mesentery containing six valves (V1–V6). When cannulated and pressurized at body temperature, these vessels resume their dynamic contractile function without any outside stimulus. Pressure was measured using a servo-null micropipette in either the first or second lymphangion (pressure measurement site, *p*_1_), and diameters were measured by edge detection as indicated by “tracking site.” Inlet and outlet pressures *p*_in_ and *p*_out_ were imposed by the heights of the inlet and outlet reservoirs, respectively. The height of the fluid in the bath above the centreline of the vessel resulted in an external pressure *p*_e_ of 0.5 cmH_2_O. In the experiments reported here, all lymphangions contracted in an essentially synchronous manner, in that time delays were small enough that intermediate valves always remained open, and a single relaxation phase (thus suction period) was detected along the entire vessel.

Discovery of subatmospheric tissue pressures by Arthur Guyton in 1963 challenged the traditional hydrostatic theory of lymph formation. This led Guyton and others to propose a ‘suction theory’ as a means of clearing lymph from these tissue beds^1^. It is now widely accepted that pressure in a variety of tissues is subatmospheric, irrespective of the method used to measure interstitial pressure^2–4^. This is of particular importance to lymph dynamics in all of the three largest lymphatic networks in the body, the subcutaneous, gastrointestinal and respiratory tissue networks^5^. Hydrostatic pressure in the initial lymphatics varies slightly around that of the surrounding tissues^2,6^ but the average pressure may be negative^6^, zero^4^, or slightly positive^7^ relative to atmospheric. Lymph formation, i.e., transport of interstitial fluid from subatmospheric tissue beds to lymphatic capillaries, is highly dependent on the pressure difference between the tissue and the initial lymphatics (Fig. 1A). In all fluid flow situations, it is the pressure difference from one location to another that drives flow, not the value of pressure at any single location. This basic mechanical fact is crucial for understanding the mechanisms behind suction.

Once inside the initial lymphatics, further movement of this fluid onward into collecting lymphatics involves overcoming adverse axial pressure differences. Initial lymphatics do not actively contract in most animals. Downstream, collecting lymphatics possess one-way valves, and the vessel segment between two valves is referred to as a lymphangion. By means of muscle in the vessel walls, lymphangions actively contract, but contraction can only increase pressure locally. This performs the important task of propelling fluid downstream. The collecting lymphatics thus propel lymph centrally against prevailing pressure gradients, and often gravity, in a process known as intrinsic pumping^8^. Relative motion of tissues surrounding lymphatic vessels, owing to events such as skeletal muscle contraction, also facilitates lymph propulsion in many tissues; this process is extrinsic pumping^9–11^. However, by itself, neither contraction nor external squeezing can pull fluid in from upstream.

With his suction theory, Guyton sought to explain how lymph could be drawn into initial lymphatics and propelled centrally. He proposed that local tissue movement transiently increases interstitial pressure sufficiently to drive fluid through the initial lymphatics^12^. This hypothesis was never proven, and measurements published since then (summarised below) fail to demonstrate any such transient interstitial pressure spikes. This fundamental question generated heated debates among researchers in the field which were never resolved, despite the significance of the issue for both physiology and diseases such as oedema, cancer, and obesity.

In the years following Guyton’s publication of the lymphatic suction theory, he and others sought experimental evidence for his ideas, but still were unable to explain the underlying mechanisms. Measurements in bat wings demonstrated the existence of transient favourable pressure differences across the initial lymphatic wall^4,13^. However, lymphatic structures in bat wings are fundamentally different from those in most other mammals in that the initial lymphatics exhibit active contractility. More recently, internal pressures as low as −50 mmHg were recorded *in situ* in initial lymphatics in the mouse thoracic wall and diaphragm^6,14,15^. Pleural pressure remained higher but still subatmospheric, so while these results demonstrate conditions favourable to lymph formation, they do not support Guyton’s theory. This is because the favourable pressure difference across the initial lymphatic wall was created by a drop in intralymphatic pressure (conclusion from our study), rather than an increase in pleural pressure as required by Guyton’s theory. The discovery and confirmation of primary lymphatic valves that prevent fluid captured by initial lymphatics from leaking back into interstitial spaces provided evidence of a further necessary passive component of lymph formation^16–19^. The primary valve system permits lymph flow into the initial lymphatics under favourable pressure differences, and prevents it from leaving when the pressure difference returns to adverse. All of the above studies were conducted in initial lymphatic *in situ* preparations. The first documented evidence demonstrating a suction mechanism in collecting lymphatic vessels was an earlier study of ours using isolated bovine mesenteric lymphatics^15^. All of these experimental approaches suffered the limitation of not being able to measure simultaneously all of the biomechanical phenomena involved in generating suction.

Here we address the ambiguities in the lymphatic suction theory by combining *ex vivo* experiments and mathematical modelling. We carried out pressure-controlled experiments to measure isolated collecting lymphatic pumping and developed a new technique to vary upstream resistance readily and quickly. This allowed us to characterize the system under conditions that closely mimic *in vivo* suction conditions. These data are augmented by mathematical modelling techniques that faithfully reproduce the experimental conditions and, more importantly, provide critical estimates of flow parameters that cannot be measured, such as dynamic pressure and flow rate distributions. We further studied the possibility that suction could be transmitted from contracting lymphangions to passive vessels upstream, in order to assess the significance of suction in lymph formation, the fundamentals of which have remained poorly understood.

This work builds on more than 50 years of research and countless debates on lymphatic suction theory. The existence of subatmospheric tissue pressures is no longer controversial, as there is ample support in the form of multiple measurements, using various techniques in different species and locations including bat wing^4^, cat mesentery^2^, rat mesentery^7^, and rat diaphragm^6,14^. However, the mechanisms by which these pressures are generated and potentially maintained by the removal of interstitial fluid via the lymphatics are not well understood. In particular, the role of phasically contracting collecting lymphatic vessels for pulling fluid via suction from the upstream lymphatic network has not been systematically studied. The body of work described in this paper provides a mechanistic explanation of lymphatic suction, and reveals the conditions necessary for it to exist. These underlying principles are important to lymph formation and flow generation under all conditions, including oedema.

## Results

### Existence and mechanisms of suction

The crucial action that facilitates drawing of fluid from tissues with subatmospheric pressure is a transient dip in intralymphatic pressure that occurs just after active collecting vessel contraction. This only occurs under certain physical conditions. In a series of experiments with isolated rat mesenteric lymphatic vessels (*N* = 5) each containing 4 to 6 lymphangions (Fig. 1B), we observed that the pressure in the first upstream lymphangion dipped below the inlet pressure early in diastole. Note that lymphatic systole and diastole have been previously defined by the start and end of the phasic lymphatic muscle contraction and are related to the measurable changes in diameter^20^.

**Figure 2:**
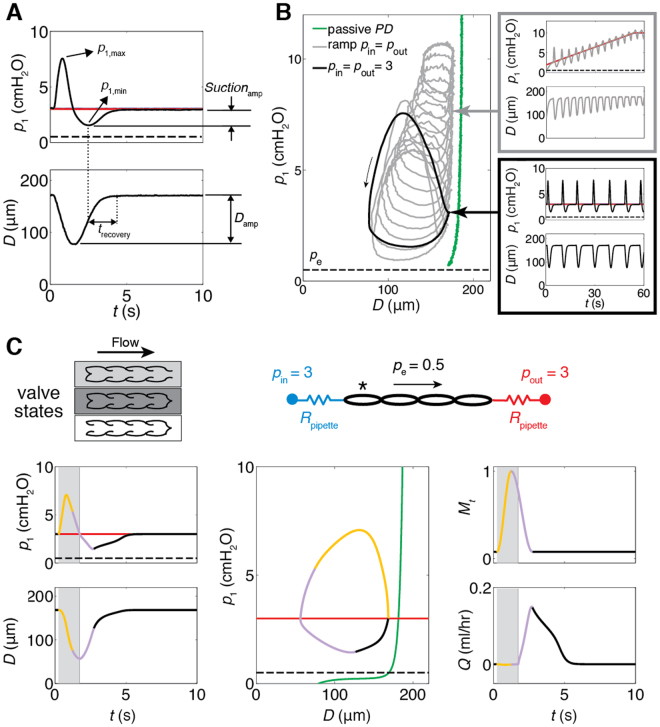
Demonstration of suction (number of experiments (*N* = 5), results shown for one experiment). (**A**) Experimental measurement of pressure and diameter traces for *p*_in_ = *p*_out_ = 3 cmH_2_O (red line) during one contractile cycle. As the vessel expands, pressure falls transiently below the upstream reservoir pressure. *Suction*_amp_ is the pressure difference between inlet pressure *p*_in_ and *p*_1,min_. *t*_recovery_ is the time constant of the fitted curve from *p*_1,min_ to end-diastolic diameter (EDD). Diameter amplitude, *D*_amp_ = *D*_max_−*D*_min_. The external pressure is shown as the dotted line on all pressure traces in this figure. Note that pressure traces for *p*_1_, *p*_a_ and *p*_b_ all indicate positive transmural pressure. (**B**) Pressure (*p*_1_) vs. diameter (left). Passive pressure-diameter curve (green) obtained in Ca^2+^-free solution. The black loop corresponds to contraction at *p*_in_ = *p*_out_ = 3 cmH_2_O (*p*_1_ and *D* traces at right, bottom), grey loops show contractions in response to a ramp increase in *p*_in_ = *p*_out_ (*p*_1_ and *D* traces at right, top). Each loop corresponds to a contractile cycle. For example, there are 14 grey loops (and 14 contractile waves shown in the traces shown at upper right of Fig. 2B) because there were 14 contractile cycles during the pressure ramp. (**C**) Note that the valve state with both inlet and outlet valve closed occurs only briefly before beginning of suction. Predictions of pressure (*p*) and diameter (*D*) in the first lymphangion, and flow rate (*Q*) through the first valve from mathematical modelling. *M*_*t*_ is the time-dependence of tension activation. Yellow, purple, and black correspond to contraction, relaxation, and diastole, respectively, derived from this curve and superimposed onto other curves. The decay of muscle activation corresponds to the purple (“relaxation”) region of the *M*_t_ curve. Background shades correspond to three valve states as indicated at top left (the isovolumetric state, dark grey, is very short). Predicted *p*_1_ and *D* closely resemble experimental results. Under these pressure conditions, flow through the first valve only occurred during suction. The timing of flow through downstream valves can be seen in Fig. 4D.

The magnitude of the dip (suction pressure amplitude, *Suction*_amp_, Fig. 2A) ranged from 0.9 to 1.5 cmH_2_O in the presence of a positive transmural pressure of 2.5 cmH_2_O. Transmural pressure (Δ*p*_tm_) is defined herein as the average of upstream and downstream reservoir pressures, minus external pressure. Upstream and downstream reservoir pressures were set to 3.0 cmH_2_O in this case, and external pressure (*p*_e_) was 0.5 cmH_2_O based on the height of the liquid in the bath surrounding the vessel. Transmural pressures of a few cmH_2_O as applied here are typical for these rat mesenteric vessels^20^. Note that it is the pressure differences that are relevant here – not individual pressure values. The recovery back to the inlet pressure was also characterized by the time constant *t*_recovery_ of an exponential curve fitted to the diameter versus time trace during diastole, which ranged from 0.47 to 0.81 s (Table 1). The average duration of contraction/relaxation cycles over the five vessels was 8.12 s; this is in line with isolated rat vessel experiments under similar conditions^21,22^. Here, the duration ranged from 4.9 to 11.2 s. The pressure waveforms showed maximum systolic pressures (*p*_max_) ranging from 5.4 to 8.8 cmH_2_O. Diastolic diameters in these vessels were 158 to 193 μm, and the amplitude of diameter change (*D*_amp_) ranged from 76 to 109 μm, indicating the typically strong contractile function of these vessels. Pressure-diameter measurements in Ca^2+^-free medium (passive vessel) indicated strongly strain-stiffening behaviour (Fig. 2B, green curve), also typical for these vessels. Suction persisted as the upstream and downstream reservoir pressures were raised in concert from 1 to 10 cmH_2_O (Fig. 2B, grey curves).

**Table 1:** Experimentally measured pumping parameters. Average ± standard deviation for seven contractions of each vessel is reported for *N* = 5 vessels for inlet and outlet reservoir pressures, *p*_in_ = *p*_out_ = 3, and external pressure, *p*_e_ = 0.5 cmH_2_O. *Suction*_*amp*_ is the pressure difference between inlet pressure, *p*_in_ and *p*_1,min_. *t*_recovery_ is the time constant of the fitted curve from *p*_1,min_ to *D*_max_.

	*Suction*_amp_ (cmH_2_O)	*t*_recovery_ (s)	*D*_amp_ μm	*D*_max_ μm	*p*_max_ (cmH_2_O)	*f* (Hz)
Vessel 1	1.4 ± 0.1	0.52 ± 0.02	98 ± 2	164 ± 1	8.76 ± 0.27	0.11 ± 0.02
Vessel 2	1.5 ± 0.1	0.47 ± 0.01	95 ± 1	172 ± 1	7.63 ± 0.06	0.10 ± 0.01
Vessel 3	1.5 ± 0.1	0.58 ± 0.02	114 ± 2	193 ± 1	5.41 ± 0.10	0.20 ± 0.01
Vessel 4	1.2 ± 0.1	0.52 ± 0.02	76 ± 1	158 ± 1	5.35 ± 0.14	0.18 ± 0.05
Vessel 5	0.9 ± 0.1	0.81 ± 0.07	110 ± 3	167 ± 3	6.09 ± 0.09	0.14 ± 0.05

Our mathematical model of contraction of lymphangions in series initially predicted the suction effect independently, and motivated the series of experiments presented here. Mathematical modelling also provided further insight into the suction mechanism, and indicated the importance of a positive transmural pressure in generating suction. The model accounts for strain-stiffening behaviour of the passive vessels under Ca^2+^-free conditions, including the transition to the highly compliant state at lower positive transmural pressures (Fig. 2C, middle panel, green curve). Vessel contractions in the model are imposed in part via *M*_t_ (the normalised time-dependent waveform of activation of lymphatic muscle cells). Total active force also depends on diameter via a length-tension relation *M*_d_. This nonlinear relationship between diameter and active tension results in the vessel reaching its minimum diameter after *M*_t_ peaks. Contraction initially increases the internal mid-lymphangion pressure *p*_1_, but this pressure falls off as fluid is pushed downstream, with the rate of decrease related to viscous loss in the first lymphangion and the downstream impedance. In the model, which does not directly incorporate external vessel tethering, positive transmural pressure (Δ*p*_tm_ > 0) was required to distend the vessel back open following muscular contraction, providing the transient energy input required for the suction effect. Vessel expansion began during the decay of muscle activation (purple portion of *M*_*t*_ curve) and the resulting pressure drop in the first lymphangion (*p*_1_ < *p*_in_) opened the inlet valve (background shading: white) (Fig. 2C). The positive transmural pressure enabled further vessel expansion during the diastolic period (black portion of *M*_*t*_ curve), resulting in continued diastolic filling. The work executed during suction to accomplish vessel refilling was 0.063 dyn cm.

### Increase in upstream resistance enhances suction effect

**Figure 3:**
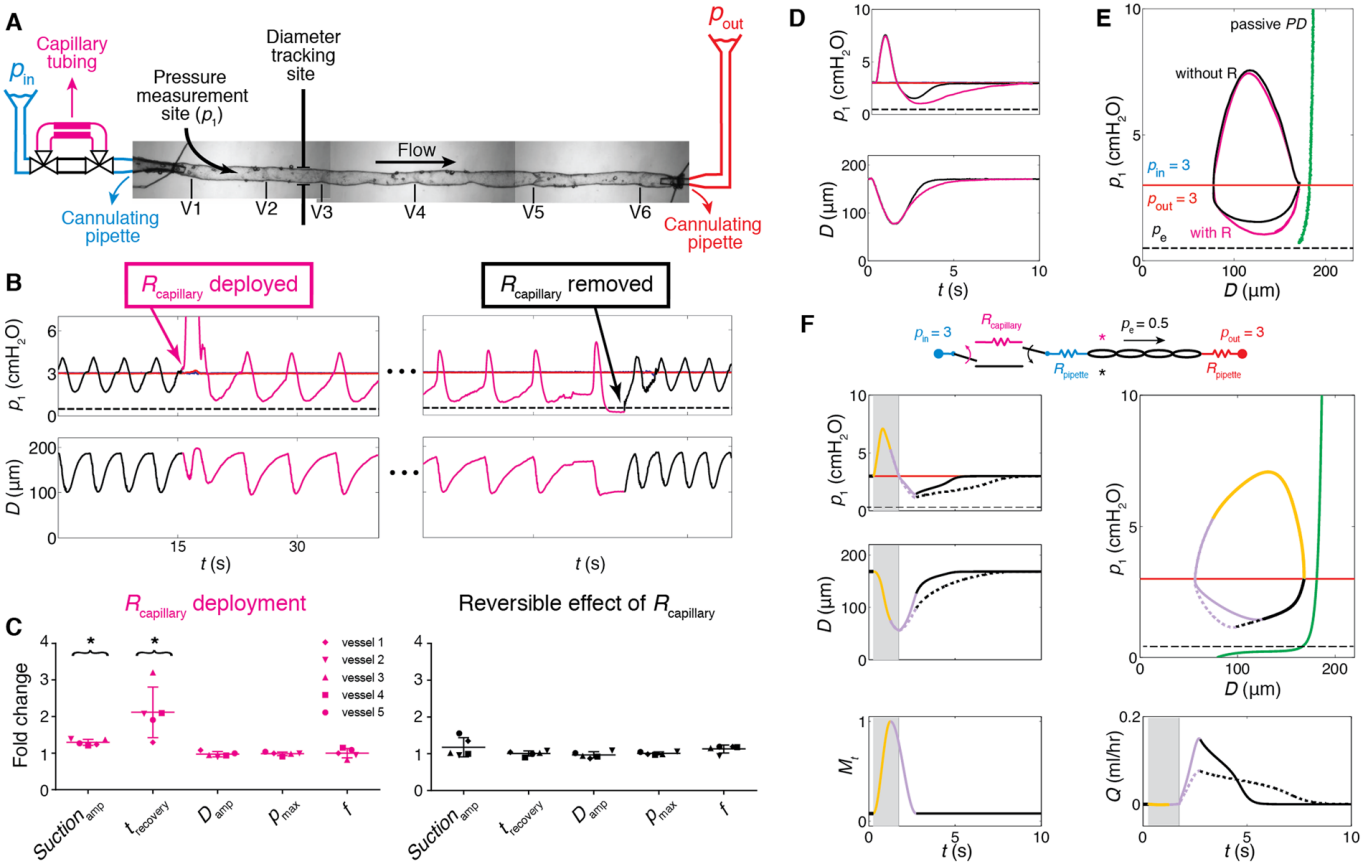
Effect of increasing upstream resistance by 2.6× (N = 5). (**A**) To simulate the effect of non**-**contracting segments (e.g., pre**-**collectors or initial lymphatics) upstream of a collecting vessel, a small-diameter section of tubing presenting a high resistance (*R*_capillary_, magenta) was inserted just upstream of the first lymphangion. Valves at both ends allowed instantaneous deployment/removal of *R*_capillary_. (**B**) Pressure and diameter traces for *p*_in_ = *p*_out_ = 3, *p*_e_ = 0.5 cmH_2_O, before (black) and after (magenta) *R*_capillary_ was deployed (left). Restoration of baseline pressures was observed following removal of *R*_capillary_ (right). (**C**) Relative change in pumping parameters when *R*_capillary_ was deployed (*N* = 5, *p*_in_ = *p*_out_ = 3, *p*_e_ = 0.5 cmH_2_O). *Suction*_amp_ and *t*_recovery_ significantly increased. For the representative experiment shown in A, contraction frequency dropped, however this was not the case for the other four vessels. Among all five experiments *D*_amp_, *p*_max_, *f* did not show significant changes (left). Comparison of contractions before deployment and after removal of *R*_capillary_ confirmed that the effect was reversible (right). Error bars indicate standard deviation. *Significant difference from 1 (*t*-test, *p* < 0.01). (**D**) A sample contraction cycle without/with *R*_capillary_ (black/magenta, respectively). (**E**) Overlap of pressure**-**diameter loops during one contractile cycle. (**F**) Predictions of the effect of *R*_capillary_ (dotted lines) from mathematical modelling. In agreement with experimental findings, *Suction*_amp_ and *t*_recovery_ increased, resulting in a longer filling phase. The chain of lymphangions maintained its time-average flow rate (<0.4% change). Background shadings refer to the valve states presented in Fig. 2C.

*Suction*_amp_ immediately and significantly increased when a small-diameter tube segment with high resistance (*R*_capillary_) representing the resistance of the upstream network of initial lymphatics was deployed upstream of the contracting vessel (Fig. 3A). Inserting this tube increased the upstream resistance by 2.6×, relative to the resistance of just the upstream pipette. Under normal conditions (without *R*_capillary_), the pressure recovered back to the baseline value well before the end of the cycle. With *R*_capillary_, the time required for pressure recovery was prolonged, but it still occurred before the end of diastole. The additional upstream resistance resulted in, on average, a 1.3× increase in *Suction*_amp_ and a 2.1× increase in *t*_recovery_. However, there were no significant changes in parameters of contractility, *D*_amp_, *f* (contraction frequency), and *p*_max_. The increased *t*_recovery_ still only covers early diastolic relaxation, and thus has minimal effects on *f*, as demonstrated in Fig. 3C. For the representative vessel (vessel 2) shown in Fig. 3A, contraction frequency decreased, but this was not the case for the other four vessels. No statistically significant change in contraction frequency occurred in any of the five experiments. For the representative vessel, *Suction*_amp_ increased from 1.5 to 1.9 cmH_2_O and *t*_recovery_ doubled, while all other parameters remained unchanged (Fig. 3D,E). The effects of the additional upstream resistance that led to increased *Suction*_amp_ and *t*_recovery_ were reversible; that is, when *R*_capillary_ was removed, both parameters returned to their initial values in all five vessels (Fig. 3C).

Our mathematical model predicted the effect of upstream resistance on suction amplitude and in fact motivated the series of experiments with *R*_capillary_. In agreement with experimental findings, lymphangion pressures dipped considerably lower than *p*_in_ during relaxation when *R*_capillary_ was included in the model, creating a 1.2× larger *Suction*_amp_ (Fig. 3F). Slower vessel expansion resulted in a prolonged period of suction and longer recovery time, again in close agreement with experiments. The work executed by suction increased by 14.5% relative to its baseline value, to reach 0.071 dyn cm. Peak flow rate dropped by almost 50%, but forward flow continued over a longer period. As a result, the vessel maintained its average flow-rate output (less than 0.4% change) despite the higher input resistance.

### Suction effect persists along the length of the vessel

**Figure 4:**
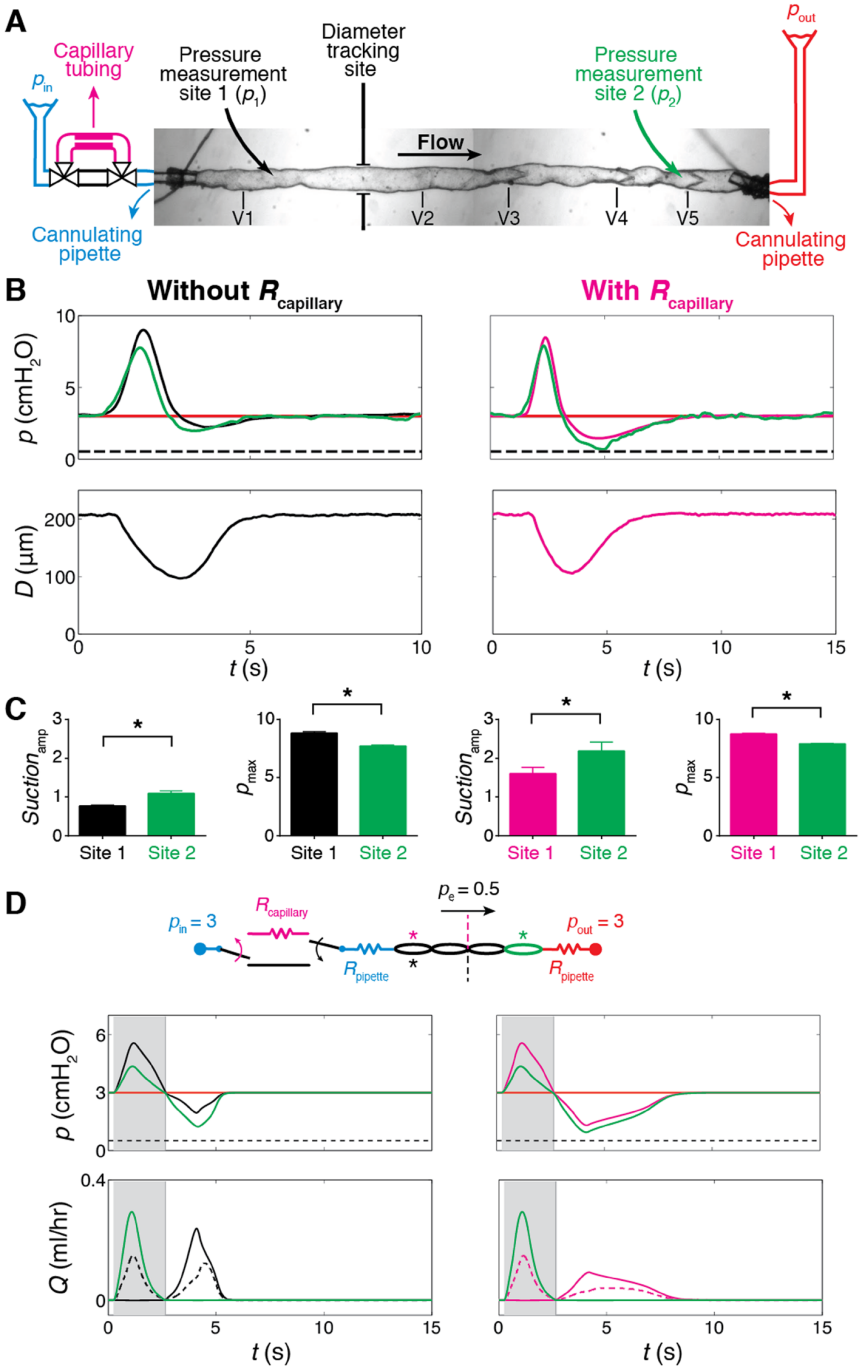
Suction persisted along the length of the lymphatic vessel (*N* = 1). (**A**) Pressure was measured at two locations: *p*_1_, downstream of V1 (black), *p*_2_, upstream of V5 (green), *D* was measured at the indicated site. (**B**) Left: traces of *p*_1_ (black), *p*_2_ (green), and *D* (for *p*_in_ = *p*_out_ = 3, *p*_e_ = 0.5 cmH_2_O). Right: traces of *p*_1_ (magenta), *p*_2_ (green), and *D*, with *R*_capillary_ upstream. (**C**) *Suction*_amp_ increased while *p*_max_ dropped at *p*_2_ with or without *R*_capillary_ upstream. Error bars indicate standard deviation. *Significant difference from measurement at site 1 (*t*-test, *p* < 0.01). (**D**) Predictions from mathematical modelling for *p*_in_ = *p*_out_ = 3, *p*_e_ = 0.5 cmH_2_O. Left (without *R*_capillary_): traces of *p*_1_ (black), *p*_2_ (green), and *Q*_1_ (flow through first valve, black), *Q*_3_ (flow through middle (third) valve, black dashed line), *Q*_5_ (flow through last (fifth) valve, green). Right: traces of *p*_1_ (magenta), *p*_2_ (green), and *Q*_1_ (magenta), *Q*_3_ (magenta dashed line), *Q*_5_ (green), with *R*_capillary_ upstream. Background shadings refer to the valve states presented in Fig. 2C. Flow profiles indicated that inlet valve (bottom panel - left: without *R*_capillary_, *Q*_1,_ black, right: with *R*_capillary_, *Q*_1,_ magenta) exclusively opens during suction, whereas the outlet valve (bottom panel - left: without *R*_capillary_, *Q*_5_, green, right: with *R*_capillary_, *Q*_5_, green) is shut during that period and only opens when upstream lymphangions are contracting.

Simultaneous pressure measurement at a second, downstream site (*p*_2_) confirmed that suction persists along the length of the vessel (Fig. 4A). Because of the viscous losses associated with antegrade flow, *p*_max_ decreased by 12.5%, while *Suction*_amp_ increased by 41% from site 1 to site 2. With *R*_capillary_ upstream, *Suction*_amp_ at both sites doubled, remaining greater at the second measurement site (Fig. 4B,C). In accordance with these experimental findings, the mathematical model predicted lower peak and minimum pressures (larger *Suction*_amp_) in downstream lymphangions. Flow through the first valve (Fig. 4D bottom panels, left: without *R*_capillary_, black, right: with *R*_capillary_, magenta) occurs solely during suction. Flow through the outlet valve, on the other hand, occurs during contraction of upstream lymphangions (Fig. 4D bottom panels, left: without *R*_capillary_, green, right: with *R*_capillary_, green). Outflow stops when pressure in the last lymphangion drops below *p*_out_ during muscle relaxation (beginning of suction) (Fig. 4D, top panels). Intermediate valves (Fig. 4D, bottom panels), however, remain open throughout the entire cycle thanks to synchronized contraction of the lymphangions in this chain and valve bias to remain open^23^. For these valves, the first peak in flow rate occurs during contraction, while the second peak is associated with suction-induced filling upon relaxation (Fig. 4D bottom left panel, black dashed line). When *R*_capillary_ is deployed (Fig. 4D bottom right panel, magenta dashed line), the outflow waveform of the vessel during contraction remains unchanged, but the incoming flow waveform through the first valve is broadened over time as previously explained. The diastolic flow waveform through the intermediate valve is similarly broadened.

### Evidence of suction in upstream vessels

**Figure 5:**
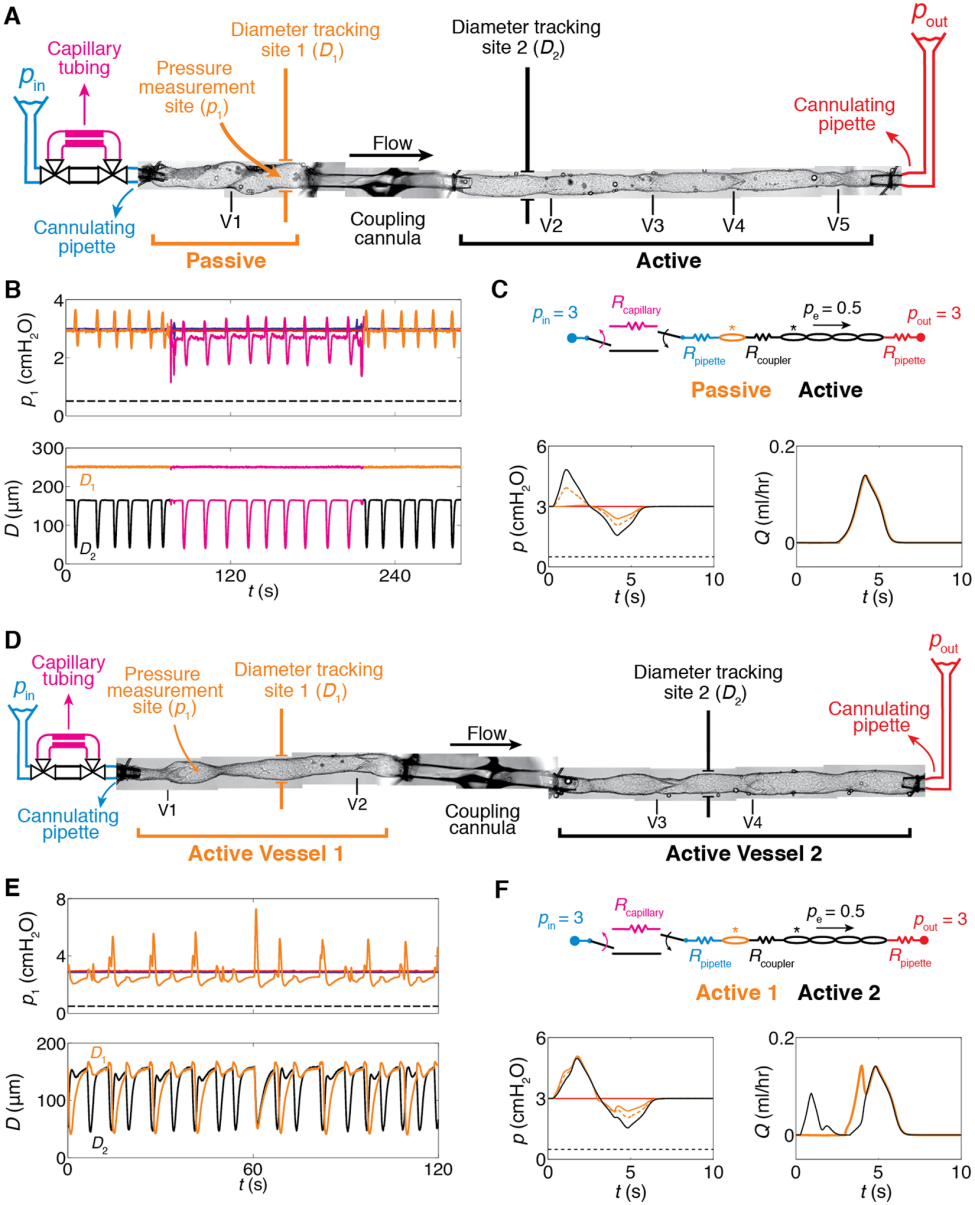
Suction from contracting lymphatic vessels is transmitted upstream to both contracting and non**-**contracting vessels. (**A**) Two lymphatic vessels were connected using a coupling cannula. Upstream vessel: mouse iliac efferent lymphatic (passive: non**-**contracting) (orange), downstream vessel: rat mesenteric lymphatic (active: contracting). Pressure (*p*_1_) was measured downstream of V1 in the passive vessel (*N* = 1). (**B**) Top: trace of *p*_1_ (orange). Bottom: *D* obtained at diameter tracking site A (orange) and B (black). Effect of *R*_capillary_ upstream (magenta). (**C**) Pressure and flow predictions from mathematical modelling. Left: pressure between two valves in the passive vessel (orange, solid), downstream of the second valve in the passive vessel (orange, dotted), pressure in the first lymphangion in the active vessel (black). Right: flow waveform in the passive vessel (orange), and through the first valve in the active vessel (black). Pressure drop from suction opened the valve in the passive segment and allowed lymphatic filling. (**D**) Two contracting lymphatic vessels from rat mesentery were connected (both active). Pressure (*p*_1_) was measured downstream of V1 in vessel 1. (**E**) Top: trace of *p*_1_ in vessel 1. Bottom: *D* obtained at diameter tracking site A (orange) and B (black). The pressure trace showed two dips in pressure associated with suction during expansion of the two vessels (*N* = 1). (**F**) Pressure and flow predictions from mathematical modelling. Left: pressure between two valves in vessel 1 (orange, solid), downstream of the second valve in vessel 1 (orange, dotted), pressure in the first lymphangion in vessel 2 (black). Right: flow waveform through the first valve in vessel 1 (orange), and through the first valve in vessel 2 (black).

*In vivo*, an actively contracting collecting vessel would be connected upstream to non-contracting initial lymphatics. Since it is not technically feasible to isolate initial lymphatics attached to a collecting lymphatic, we inserted a passive (non-contracting) vessel upstream of the contracting vessel to determine to what extent the suction pressure from a collecting lymphatic would be transmitted upstream (Fig. 5A–C, 5B experimental results, 5C modelling results). Diameter measurements confirmed the lack of active contraction in the passive vessel, whereas pressure within it varied dynamically as the downstream active vessel contracted, and dipped below the inlet pressure at a suction amplitude of approximately 0.5 cmH_2_O. (The lack of change in diameter in response to changes in pressure indicates that the passive vessel is operating in the relatively stiff part of the pressure-diameter curve under these conditions.) Insertion of the high-resistance capillary tubing upstream of the passive vessel increased the suction amplitude to >1 cmH_2_O. Thus, all features of suction in contracting vessels are transmitted upstream to the non-contracting vessels. Pressure traces at multiple locations (from mathematical modelling) revealed a smaller *Suction*_amp_ and lower *p*_max_ in the passive segment compared to the active vessel. Notably, *Suction*_amp_ created a sufficient pressure drop to open the valve in the passive segment and permit diastolic filling of the two connected vessels (Fig. 5C).

In the vast majority of isolated vessel experiments conducted (including all of those done for this study in rats), the lymphangions effectively contract synchronously. Because of the strong electrical coupling, propagation speed is high and thus the time delay between subsequent lymphangions (each approximately 1 mm in length) is low, as described earlier. We therefore performed a series of experiments targeted at this issue, as shown in Fig. 5D,E. In this case, a rat mesenteric lymphatic vessel was split into two parts that were hydraulically connected but electrically isolated with a short glass pipette. The vessels exhibited asynchronous contractions relative to one another. The corresponding diameter and pressure data from the experiments are shown in Fig. 5E. We provide corresponding pressure data, as well as flow data not available experimentally, from mathematical modelling (Fig. 5F).

We identified two distinct suction dips, associated with the separate expansion of each vessel, when the two active segments were connected. Traces of pressure and diameter measured in vessel 1 (orange) together with diameter in vessel 2 (black) demonstrated that the first period of suction occurred during expansion of vessel 1, whereas a second suction dip developed during expansion of vessel 2, with vessel 1 producing the greater *Suction*_amp_. Notably, at the instant when the two vessels synchronized briefly (just after *t* = 60 s), only one suction dip was detectable. In this interval of synchronized contraction, *Suction*_amp_ and *p*_max_ were both elevated (Fig. 5E). Mathematical modelling predicted one suction interval in vessel 2 (with greatest *Suction*_amp_) for a scenario where vessel 2 contracted with slight time delay relative to vessel 1. Diastolic filling through the first valve (vessel 1, orange) occurred during suction of vessel 1 and vessel 2 (peak 1 and peak 2, respectively). In vessel 2 (black), forward flow was maintained during contraction of vessel 1 (peak 1) and expansion of vessel 2 (peak 2) (Fig. 5F).

### Effects of transmural and axial pressure differences

**Figure 6:**
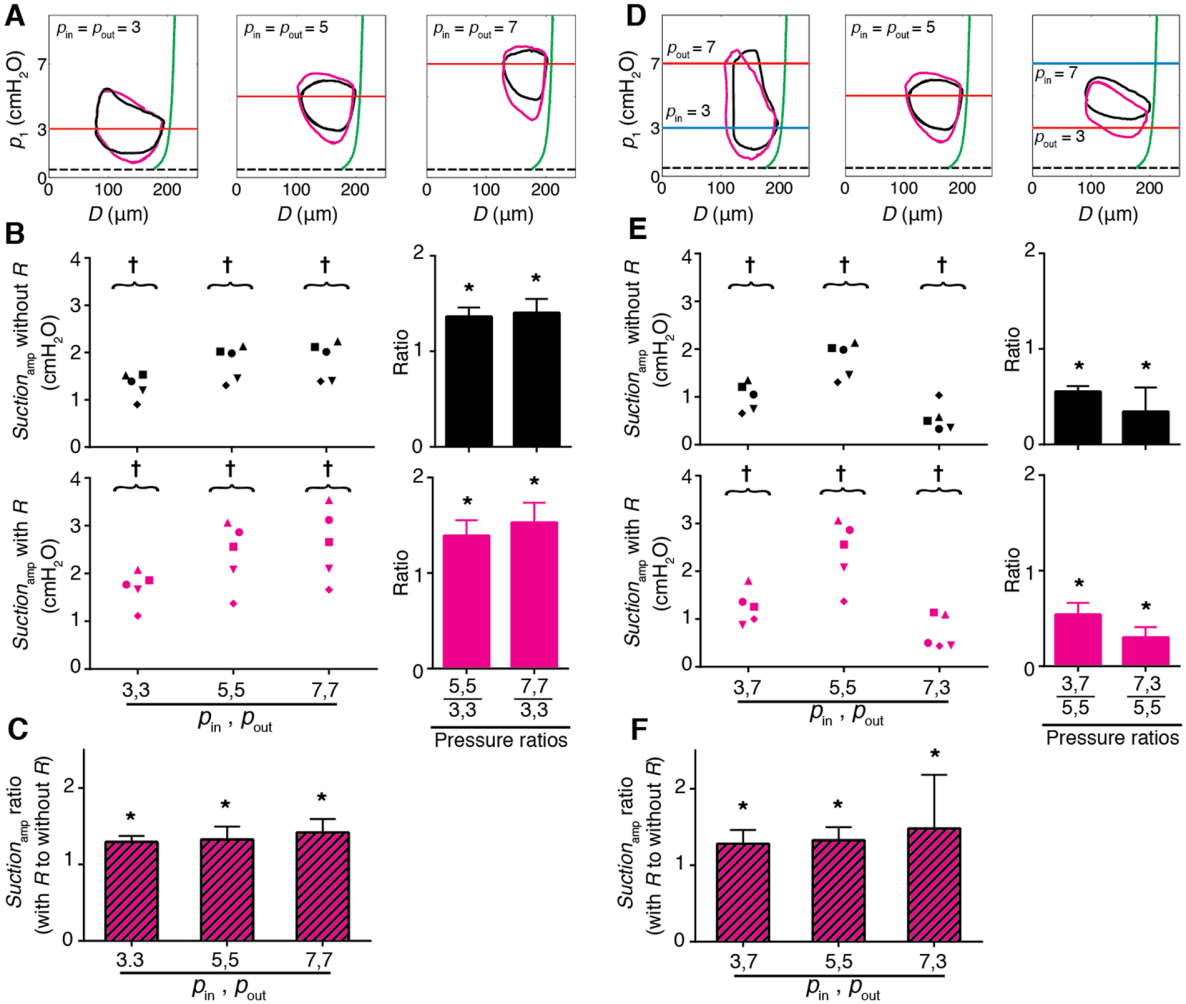
Effect of transmural and axial pressure differences (*N* = 5). (**A**) Representative pressure vs. diameter in a single contraction cycle without *R*_capillary_ (black) and with *R*_capillary_ upstream (magenta) for *p*_in_ = *p*_out_ = 3, 5, 7 cmH_2_O (left to right). Passive pressure**-**diameter curve is in green. (**B**) Top: without *R*_capillary_. Average *Suction*_amp_ at *p*_in_ = *p*_out_ = 3, 5, 7 cmH_2_O (*N* = 5, *p*_e_ = 0.5 cmH_2_O) (left). †Significant difference from 0 (*p* < 0.01). Relative increase in *Suction*_amp_ at *p*_in_ = *p*_out_ = 5, 7 cmH_2_O relative to *p*_in_ = *p*_out_ = 3 cmH_2_O averaged for five vessels (right). *Significant difference from 1 (*p* < 0.01). Bottom: same plots with *R*_capillary_ upstream. (**C**) Relative increase in *Suction*_amp_ at *p*_in_ = *p*_out_ = 3, 5, 7 cmH_2_O following deployment of *R*_capillary_ and averaged for five vessels. †Significant difference from 0 (*p* < 0.01). (**D**) Representative pressure vs. diameter in a single contraction cycle without *R*_capillary_ (black) and with *R*_capillary_ upstream (magenta) for *p*_in_, *p*_out_ = (3, 7), (5, 5), (7, 3) cmH_2_O (left to right). (**E**) Top: without *R*_capillary_. Average *Suction*_amp_ at *p*_in_, *p*_out_ = (3, 7), (5, 5), (7, 3) cmH_2_O (*N* = 5, *p*_e_ = 0.5 cmH_2_O) (left). †Significant difference from 0 (*p* < 0.01). Fold change in *Suction*_amp_ at *p*_in_, *p*_out_ = (7, 3), (3, 7) cmH_2_O relative to *p*_in_ = *p*_out_ = 5 averaged for five vessels (right). †Significant difference from 0 (*p* < 0.01). Bottom: same plots with *R*_capillary_ upstream. (**F**) Fold increase in *Suction*_amp_ at *p*_in_, *p*_out_ = (3, 7), (5, 5), (7, 3) cmH_2_O following deployment of *R*_capillary_ averaged for five vessels. Error bars indicate standard deviation. *Significant difference from 1 (*t*-test, *p* < 0.01).

Variation of transmural [(*p*_in_ + *p*_out_)/2 − *p*_e_] and axial pressure differences (∆*p* = *p*_out_ − *p*_in_) influenced *Suction*_amp_. Increasing the transmural pressure difference enhanced *Suction*_amp_. Raising inlet and outlet pressures in concert (*p*_in_ = *p*_out_) from 3 to 5 and then 7 cmH_2_O resulted in 1.36× and 1.4× increases in *Suction*_amp_ respectively, relative to that with *p*_in_ = *p*_out_ = 3 cmH_2_O. With *R*_capillary_ upstream, suction increased by 1.39× and 1.53× at 5 and 7 cmH_2_O, respectively, relative to that with *p*_in_ = *p*_out_ = 3 cmH_2_O. At all transmural pressure differences, deployment of *R*_capillary_ created a greater *Suction*_amp_ (Fig. 6C).

Lower *Suction*_amp_ was observed when the axial pressure difference was adverse (∆*p* > 0, thus *p*_out_ > *p*_in_). *Suction*_amp_ was also reduced by a favourable axial pressure difference (favourable: ∆*p* < 0, thus *p*_out_ < *p*_in_). Specifically, under adverse (*p*_in_, *p*_out_ = 3, 7 cmH_2_O) and favourable (*p*_in_, *p*_out_ = 7, 3 cmH_2_O) axial pressure differences, *Suction*_amp_ dropped to 0.55× and 0.34× of its value at zero axial pressure difference (∆*p* = 0, *p*_out_ = *p*_in_ = 5 cmH_2_O), respectively. Note that in the presence of a favourable axial pressure difference, *Suction*_amp_ is more properly defined as the difference between end-diastolic pressure and minimum intralymphatic pressure. Similar reductions occurred with *R*_capillary_ deployed upstream under adverse and favourable axial pressure differences (*Suction*_amp_ decreased 0.54× and 0.34×, respectively, relative to ∆*p* = 0; Fig. 6D,E). Here again, adding the upstream resistance amplified *Suction*_amp_ under all axial pressure differences (Fig. 6F). Note that different pressure boundary conditions alter the valve behaviours. The valve directions of course remain the same while the pressures are adjusted.

### Negative transmural pressure differences and vessel tethering

**Figure 7:**
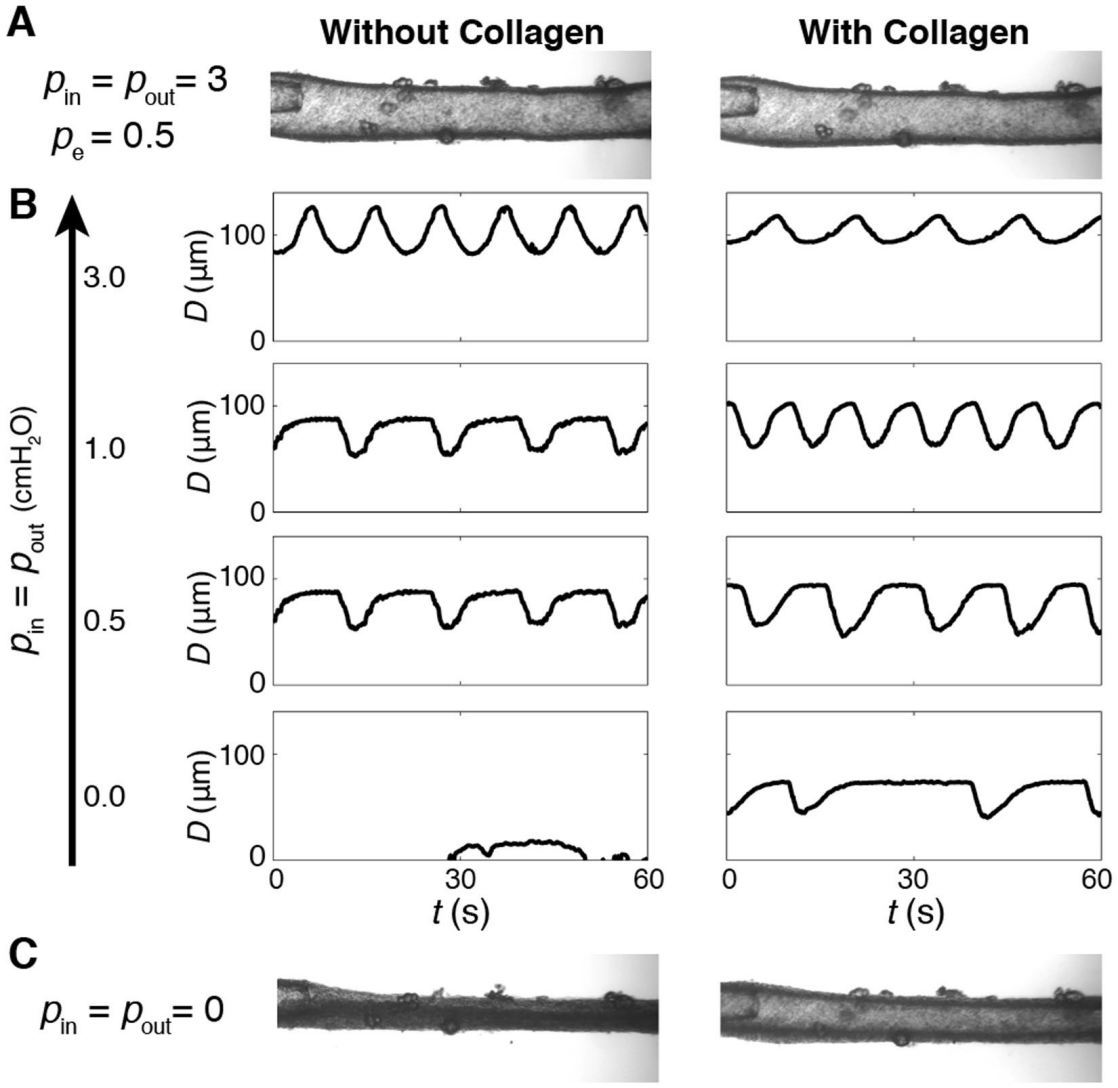
The importance of external tethering in the presence of negative transmural pressure (*N* = 1). (**A**) Left: image of isolated and cannulated mouse vessel pressurized at *p*_in_ = *p*_out_ = 3 cmH_2_O. Right: image of the same vessel at the same pressure with collagen surrounding the vessel. (**B**) Diameter traces showing contractions of isolated mouse vessel at *p*_in_ = *p*_out_ = 3, 1, 0.5, and 0 cmH_2_O (*p*_*e*_ = 0.5 cmH_2_O for all cases) (left: without collagen, right: with collagen). (**C**) At *p*_in_ = *p*_out_ = 0 (negative transmural pressure) the vessel collapsed (left), but following gelation of collagen, the vessel lumen remained open and contractions continued.

Lowering the transmural pressure difference gradually reduced *D*_amp_, until the vessel collapsed under a negative Δ*p*_tm_ = −0.5 cmH_2_O (*p*_in_ = *p*_out_ = 0, *p*_e_ = 0.5) (Fig. 7A, left) and contractions stopped. Gelation of collagen in the fluid bath surrounding the same vessel to simulate *in vivo* external tethering slightly lowered *D*_max_ (3−5 μm decrease) under Δ*p*_tm_ = 2.5 and 0.5 cmH_2_O, but afterwards the vessel achieved substantially larger diastolic diameters when transmural pressure dropped to 0 and **−**0.5 cmH_2_O. Additionally, the presence of collagen reduced *D*_amp_ by 40% at Δ*p*_tm_ = 2.5 but contractions were stronger (relative to no collagen) at Δ*p*_tm_ = 0.5 cmH_2_O. *D*_amp_ at Δ*p*_tm_ = 0 almost doubled after the vessel was tethered in collagen. Most importantly, external tethering with collagen enabled the vessel to contract under a negative transmural pressure (Δ*p*_tm_ = −0.5 cmH_2_O), though at this negative transmural pressure, the frequency of contractions in collagen was lower than that observed during positive transmural pressure (Fig. 7B). In these experiments, under conditions where *p*_in_ = *p*_e_ or lower, during suction lymphangion pressure falls below both *p*_in_ and *p*_e_.

## Discussion

The publications by Guyton and others, although transformative in their time, only speculated about the role of the lymphatic system in removing fluid from tissue beds at subatmospheric pressures. No data were provided in any of those publications to support the speculation or prove Guyton’s suction hypothesis. Since that time there has been support for the concept that the extrinsic pumping mechanisms of lymphatics associated with the respiratory system can indeed generate suction along the lymphatics^6,14^ However, these studies were conducted in the unique extrinsic lymph pump in the respiratory system and were made in reference to generating the “negative pressure gradients” needed to fill the initial lymphatics.

While Guyton and others speculated on potential mechanisms related to the lymphatic system, none of those studies considered the potential importance of the collecting lymphatics in generating suction. Collecting lymphatics are structurally and functionally very different from initial lymphatics. Initial lymphatics have no internal valves and do not actively contract. Notably, initial lymphatics are tethered on their abluminal surfaces to adjacent tissues^9^, creating openings between initial lymphatic endothelial cells when interstitial pressure rises (“primary valves”), which facilitate lymph formation. Our results are focused on the collecting lymphatics that actually perform the intrinsic pumping function. To the degree that such work can be characterised by a hypothesis, it would be that collecting vessel relaxation after contraction is the key mechanism that allows lymphatics to pull fluid from tissues at subatmospheric pressure, and that positive transmural pressure or external tethering is required. Collecting lymphatics are certainly also tethered to the external tissue in some way (likely through integrin binding to surrounding matrix), but not in the same way as initial lymphatics. The novelty of our results is that they demonstrate the importance of positive transmural pressure or external tethering on generation of suction by collecting lymphatics and how this extracts fluid from upstream segments. This can be viewed as a passive effect, in that it does not depend directly on any active contraction of the lymphangion in which the suction occurs (although active contraction must have occurred just prior).

Our study of pumping mechanics in multi-valve collecting lymphatic vessels *ex vivo*, together with mathematical modelling, demonstrates that lymphatic vessel expansion during the diastolic phase generates the suction through the inlet valve that is required for diastolic filling in the absence of a favourable pressure gradient. Without suction-derived diastolic filling, there would be no way for the vessel to generate stroke volume on the next contraction. This is analogous to lusitropy as described for the cardiac pump. When extracting fluid from tissues at subatmospheric pressures, suction is thus a key determinant of lymphatic pumping.

Collecting vessel contraction can only raise the intralymphatic pressure, *p*_1_. Under conditions in which the pressure difference across the downstream valve *p*_1_−*p*_b_ is favourable, the valve opens and flow is propelled forward. It is physically impossible for vessel contraction to decrease intralymphatic pressure below the inlet or outlet pressures. Lowering the intralymphatic pressure below the inlet pressure (so that the pressure difference *p*_a_ − *p*_1_ > 0, necessary for diastolic filling under the conditions in which suction is observed) requires positive transmural pressure. The external pressure is independent of the upstream and downstream pressures, and depends on anatomical location, surrounding tissue, and physiologic state. Thus, if the lymphatics are functioning in a tissue with subatmospheric tissue pressure, the external pressure on the collecting lymphatic is simply negative with respect to atmospheric pressure. External pressure may still be less than or greater than the intralymphatic pressure. It is therefore important to realise that there are three imposed pressure values required to specify the mechanical environment of a lymphangion: upstream, downstream and external. The important actions of lymphatic pumping depend on pressure differences (not just gauge values of individual pressures), so if these three pressures are raised or lowered in concert, there is no change in the forces to which the vessel is subjected. The fundamental importance of pressure differences is also evident in the previous work by Negrini and Moriondo, where lymph formation and pumping are shown to be dependent on the pressure differences between the respective parts of the lymphatic tissues^6,14^.

Additionally, we provide evidence that the suction pressure generated by collecting lymphatics in the presence of positive transmural pressure is transmitted upstream, and is sufficient to extract fluid from tissue beds through networks of initial lymphatics. There is no previous quantitative proof that this was the case. Schmid-Schönbein^9^ estimated for a hypothetical tissue cylinder of 1 mm radius drained by an initial lymphatic of 10 µm radius that the pressure drop required for adequate transport of fluid from the tissue space to the initial lymphatic would be only 0.12 cmH_2_O. Furthermore, a recent study estimated the viscous pressure drop through an initial lymphatic network to range from 0.4 to 3.5 cmH_2_O^24^. These estimates indicate that the transient pressure drops during suction suffice to open the primary valves and facilitate lymph formation. The resistance provided by the upstream initial lymphatics, estimated by Sloas *et al*.^24^, was included in both our experiments and models. In isolated vessels pumping with an increased inflow resistance upstream, additional work was required for the passive re-expansion of the vessel to draw fluid in. Slower relaxation during suction did eventually result in the expansion of the vessel to its original diastolic diameter (without added resistance), enabling the vessel to maintain its average flow output. These findings demonstrate that the suction mechanism is sufficiently robust to enable the vessel to overcome the increase in upstream resistance.

The implications are that the lymphatic system has the ability both to maintain flow in the presence of changing upstream conditions, and to remove fluid from tissue beds at subatmospheric pressure. This is likely how subatmospheric tissue pressures are generated during development, although our experiments did not address that issue specifically. Guyton’s suction theory was that transient increases in tissue pressures due to adjacent tissue movement drive interstitial fluid into initial lymphatics, creating net lymph flow because of the initial lymphatic primary valves. But the theory did not explain how subatmospheric pressure could persist in the absence of tissue movement. Furthermore, dynamic pressure measurements in the diaphragm indicate continuously subatmospheric interstitial and intralymphatic pressures^6,14^. The lack of observed transient supra-atmospheric pressure spikes in the diaphragm, coupled with our results, indicates that the mechanisms behind lymphatic suction and thus subatmospheric tissue pressures are rooted in positive transmural pressure and external vessel tethering.

For chains of 4−6 lymphangions in series studied here, we observed suction during vessel relaxation in all lymphangions, with an increasing amplitude along the length of the vessel. This resulted in a favourable pressure difference across each of the intermediate valves, which were held open throughout the contractile cycle. The final outflow valve in the chain was opened only during active contraction. The inlet valve, however, only opened during suction, further illustrating that suction is required for effective filling under adverse axial pressure differences (∆*p* > 0, *p*_out_ > *p*_in_). The valve states are likely more complex *in vivo*, due to dynamic upstream and downstream pressures as well as potentially asynchronous contractions and the effect of network branch points. However, the timings of the contractions are largely irrelevant to the generation of suction. This is easily rationalised with proper recognition of the importance of pressure differences. Whether or not suction occurs in the first lymphangion in a chain is completely independent of the state of contraction of the downstream lymphangions. In the extreme case of completely asynchronous contractions (maintaining the overall pressure conditions studied here), the valve between the first and second lymphangions would close at the end of systole of the first lymphangion, which would then fill due to the suction phenomenon exactly as demonstrated here for synchronous contractions.

Suction dynamics were significantly affected by the axial pressure difference ∆*p*. For example, if the pressure upstream of the incoming valve to a chain of lymphangions is sufficiently high to create a favourable axial pressure difference (∆*p* < 0, *p*_out_ < *p*_in_), suction is not required to open the first valve, and the suction pressure amplitude is reduced. This could occur as a result of increased upstream tissue pressures, which occur in some forms of oedema^25^. However, higher upstream pressure due to elevated tissue pressure can result in negative collecting vessel transmural pressures, possibly resulting in collecting vessel collapse. This would eliminate suction, increase passive resistance, and thus reduce flow. We are not aware of any *in vivo* data of simultaneous interstitial pressure and intralymphatic pressure measurements in oedematous conditions. The possibility of collapse of collecting lymph vessels under elevated tissue pressure is a prediction based on basic mechanics, and has not been observed experimentally, to the best of our knowledge.

The potentially deleterious effects of negative transmural pressure on collecting lymphatics would be mitigated if the vessel were appropriately tethered to the surrounding tissue. This is almost certainly the case for collecting lymphatics of the diaphragm, where these vessels maintain a circular cross-section and visibly contract despite significantly subatmospheric internal pressures^6,14,26^. Unfortunately, there are no thorough characterizations of collecting vessel tethering. The anchoring filaments connected to initial lymphatic endothelial cells have not been shown to exist in collecting vessels, and in any case collecting vessel tethering would involve connections at the outer surface of the vessel rather than the endothelial cells. With effective tethering, active vessel contraction would result in elastic potential energy being stored in the surrounding tissue, which would then serve to pull the vessel back open after contraction has ceased, resulting in suction under appropriate conditions. We attempted to simulate this with our collagen gel experiments, as an open water bath is incapable of storing elastic potential energy. There was enough adhesion between the collagen gel and the vessel wall to pull the vessel back open under negative transmural pressures that collapsed the vessel without the collagen gel present. This adhesion may have resulted from surface tension or direct adhesion. We have shown that these vessels show significant expression of integrin components necessary for adhesion to collagen^27,28^.While we are uncertain of the exact adhesion mechanism, the results do clearly illustrate that external tethering facilitates the suction effect in the presence of negative transmural pressures.

Our mathematical model accounts for contractile machinery of collecting lymphatic vessels using available experimental measurements. In this model, we did not adapt the contraction frequency, or strength of lymphatic muscle cell contractions for different pressure scenarios. Given the small extent of change in these quantities in the experiments, this was not a serious limitation in modelling the suction effect. Others have suggested pumping control mechanisms involving shear stress-mediated nitric oxide and calcium production, which could have been incorporated into this model if it had been necessary^29^. Otherwise, the validity of the model has been subjected to extensive testing in the form of comparison to available experimental data and parameter sensitivity analysis^30–32^.

### Clinical Implications and Conclusions

There are several tissues in which subatmospheric pressures have been observed, including those prone to oedema. This condition provokes changes in many variables affecting interstitial and lymphatic system transport, including interstitial pressures, intralymphatic pressures, and resistances of tissues and lymphatics. These clearly have implications for all of the pressure differences shown to be central to suction generation in our results. Pathologic changes in lymphatic pumping of course require further study, but no progress is achievable without a fundamental understanding of the functioning of the healthy system. The incurable disease of lymphoedema affects millions of patients, including breast cancer survivors. Interstitial pressures in the oedematous arm following mastectomy are significantly higher (1.9 ± 2.0 cmH_2_O) compared to the control arm, where subatmospheric pressures (−2.8 ± 3.0 cmH_2_O, p < 0.01) are noted^25^. Significant alteration of interstitial fluid pressure following surgical node resection (from subatmospheric to supra-atmospheric) suggests that the suction mechanisms would be altered in oedema. Current compression therapies for lymphoedema rarely cure oedema, but rather only help as part of a comprehensive management strategy^33,34^. There is little quantitative evidence of the effects that these therapies have on lymph flow, in part because continuously monitoring pressures and flow rates *in vivo* is not feasible. Limb volume can be measured with various techniques, but in the absence of information on the intervening mechanisms affecting volume, there is little quantitative basis for improving lymphoedema care, or even comparing one compression algorithm to another. More quantitative studies of the relationships between oedematous tissue biomechanics and lymphatic pumping on a larger network scale could enhance the standard of care by identifying optimal compression parameter settings that bring about the desired physiological outcome. Our modelling results demonstrate the importance of diastolic expansion of collecting lymphatics, which would be limited by continuous externally applied compression. Treatment strategies that take advantage of suction to maximize fluid entry to the system may improve lymph flow.

Apart from regional disruption of the lymphatic system by radiation or surgery that often leads to oedema, impairment of lymphatic pump function and lymphoedema are associated with several other disease conditions. For example, lymphatic dysfunction has been reported in association with Crohn’s disease and inflammatory bowel disease, with significant reduction in contractility and lymph flow^35,36^. Available clinical data suggest a possible link between obesity and lymphatic dysfunction, which results in localized lymphoedema in morbidly obese patients^37^, associated with reduction in contraction frequency^38,39^. Decreased contraction frequency has also been observed in aging vessels^40^. Genetic conditions such as Cantú Syndrome also present with symptoms of lymphoedema^41^.

In summary, our integrated modelling and experimental framework has revealed new insights into lymphatic suction and its contribution to subatmospheric pressures in tissue beds. For several decades, these topics have been debated in the physiology and lymphatic communities. This new insight into lymphatic pumping should facilitate future studies of lymphatic transport and tissue hydrodynamics with the aim of designing better treatment methods for a host of diseases associated with lymphatic pump dysfunction.

## Materials and Methods

### Isolated vessel experiments

Vessel isolation, cannulation, pressure control, as well as diameter tracking and pressure recording were performed as previously described^23^ and are briefly summarized below. All animal protocols were approved by the Institutional Animal Care and Use Committee of University of Missouri at Columbia and conducted in accordance with the National Institutes of Health Guide for the Care and Use of Laboratory Animals.

### Vessel isolation

Male Sprague-Dawley rats (150–300 g) were anesthetized and a midline abdominal incision was performed to exteriorize a loop of duodenum for vessel dissection. Collecting lymphatic vessels with inner diameter of 80–220 μm, containing four or more valves, were identified and dissected from the mesenteric arcade. Isolated vessels were placed in physiological saline solution (PSS) containing albumin (APSS, formulated as previously described^23^) at room temperature. Mouse iliac post-nodal lymph vessels were used to test transmission of suction to upstream non-contracting vessels. In contrast to mesenteric collecting lymph vessels, mouse iliac lymph vessels do not exhibit phasic contractions, and are suited to study the effects of a non-contracting vessel upstream.

### Vessel cannulation and pressurization

Lymphatic vessels containing 4–6 valves were cleaned of fat and loose tissue. After cleaning, the vessel segment was cannulated with a glass micropipette at each end. Vessel cannulation was performed in a 3 ml chamber filled with PSS. After cannulation, the apparatus containing the cannulated vessel was placed on the stage of an inverted microscope. To ensure accurate diameter tracking at high pressures, the axial length of the unsupported vessel was adjusted to eliminate buckling at the start of the experiment with *p*_in_ and *p*_out_ temporarily set to 9 cmH_2_O via standing reservoirs. To prevent changes in osmolarity due to evaporation of the PSS in the cannulation chamber, a continuous flow of heated PSS (0.5 ml/min) was maintained outside the vessel throughout the experiment.

### Cannulating pipette resistances

Prior to these experiments, pipette flow resistances (*R*_pipette,up_ = 1.84 × 10^7^, *R*_pipette,down_ = 7.2 × 10^6^ g/cm^4^ s) were calculated from the measured pressure drop across, and flow through, the pipette. These measured resistance values were applied in the mathematical model. In addition to the inlet and outlet cannulating pipettes used in all experiments to connect the vessel to the rest of the flow loop, we simulated the effect of non-contracting elements upstream of the collecting lymphatic vessel (such as initial lymphatics) by including a higher-resistance capillary tubing (inner diameter = 150 µm, length = 8 cm, *R*_capillary_ = 4.8 × 10^7^ g/cm^4^ s) upstream of the cannulated vessel (Fig. 3A). The resistance tube was connected to three-way stopcock valves at both ends, which enabled instant addition of its resistance to, or removal from, the flow loop.

In this study the inlet and outlet pipettes were necessary to perform the experiments, and are considered as the baseline resistance that is perturbed by the addition of another resistance (*R*_capillary_) in series upstream of the vessel. Thus, the resistances upstream add together (*R*_pipette,up_ + *R*_capillary_), changing pumping behaviour in a manner that helps explain the suction phenomenon. The resistances were measured for each individual pipette. Because resistance is strongly dependent on diameter (inverse fourth power) and pipettes are handmade, the two pipettes were not identical and had different resistances. The total upstream resistance (after adding the additional resistance) was similar to that reported by Sloas *et al*.^24^, who estimated the flow resistance of an excised network of initial lymphatics leading to collecting lymphatics. Thus, the requirement for pipettes to perform these experiments simulates the conditions experienced by these vessels *in vivo*.

### Diameter recording

Microscopic vessel images were captured using a Fire-Wire CCD camera (model A641FM; Basler, Ahrensburg, Germany). Vessel inside diameter at one location was tracked using a custom algorithm throughout the experiment^42^. Low-pressure transducers (CyberSense model 104; Nicholasville,KY) were used to measure *p*_in_ and *p*_out_. Pressure signals were digitized (16-bit National Instruments a.d.c) and recorded with diameter data at 30 Hz (LabVIEW; National Instruments). Diameter tracking at additional sites along the vessel was performed in post-processing.

### Servo control of inlet and outlet pressure

After initial equilibration via standing reservoirs, control of inlet and outlet pressure was switched to a custom-made, analogue servo-control system as previously described^23,43,44^. Electromagnetic actuators (model V102; Ling Dynamic Systems, Royston, UK) were connected to the inlet and outlet pipettes and powered by a hardware-based servo-controller through unitary-gain power amplifiers. The output signal from the pressure transducers was compared to reference voltages specified by a computer running LabVIEW and pump voltages were adjusted as required^43^. The system permitted application of step- or ramp-pressure waveforms to the input and/or output of the isolated contracting vessel segment.

### Servo-null pressure recording

The servo-null method was used to measure intraluminal pressure (*p*_1_) directly, as previously described^23^. *p*_1_ was measured immediately downstream of the input valve (V1) (in Fig. 4 pressure *p*_2_ is also measured in a downstream lymphangion). The zero pressure reading was set with the servo-null pipette tip placed just outside the vessel wall. Micropuncture was performed after cannulation but before temperature was raised to 37 °C (i.e. while the vessel was not contracting) to minimize trauma. When the temperature reached 37 °C, *p*_in_ and *p*_out_ were elevated simultaneously to adjust the servo-null gain and offset to match the pipette pressures. At 37 °C and an internal pressure of 1 cmH_2_O, stable rhythmic contractions normally developed within 20–40 min.

### Vessel contraction synchrony

Previous studies with isolated vessels have demonstrated that contractions of adjacent lymphangions occur with minimal time delay, such that they are effectively synchronized. Using high-speed videomicroscopy, we have reported delays of approximately 100 ms between adjacent lymphangions in rat mesentery^45^. The average duration of a contractile cycle was 8.12 s for the vessels studied here. If the lymphangions had been contracting maximally out of phase (180 degree difference), the time delay between contractions of successive lymphangions would have been 4.06 seconds. A 100 ms delay corresponds to 4 degrees of dephasing, which in the context of lymphatic pumping is effectively in phase.

### Experimental protocol

After stable contractions were achieved, inlet and outlet pressures were gradually increased over 60 s at a constant rate from 0 to 10 cmH_2_O. Such pressure and diameter data provide estimates for tension generated by lymphatic muscle cells at different diameters and pressures for mathematical modelling (Fig. 2B). In the next step, both inlet and outlet pressures were brought down to 3 cmH_2_O, and allowed to stabilize for 2–5 min. After recording of stable contractions at this pressure for 2 min, both pressures were raised in a stepwise fashion first to 5 and then to 7 cmH_2_O (Fig. 6A–C). Following any change in inlet and outlet pressures, data were collected 2–5 minutes after the change, to ensure that contractions had stabilized. Additionally, an adverse pressure difference (*p*_in_ = 3, *p*_out_ = 7 cmH_2_O) and a favourable pressure difference (*p*_in = _7, *p*_out_ = 3 cmH_2_O) were investigated (Fig. 6D–F). These pressure scenarios were chosen to examine the effect of afterload and preload while transmural pressure remained constant on average. In all experiments, an external pressure of 0.5 cmH_2_O was imposed by the height of the fluid surrounding the vessel. Pressure values are thus quoted relative to atmospheric pressure, whereas transmural pressure is simply the internal pressure minus the external pressure of 0.5 cmH_2_O.

Passive vessel diameter was measured at different transmural pressures by ramping up inlet and outlet pressures in concert (*p*_in_ = *p*_out_) at the end of each experiment in Ca^2+^-free APSS. Ca^2+^-free solution contained 3.0 mM EDTA instead of CaCl_2_ in APSS. The functional form for our pressure-diameter relationship employed in the model corresponds to the shape of every lymphatic vessel pressure-diameter curve reported in the literature of which we are aware, spanning vessels from different anatomical locations and species. To assess the mechanical properties of any tissue, it is appropriate to isolate it from the surrounding tissues, and these are the types of measurements on which we rely. Application of those mechanical properties in a physiologic manner then requires consideration of the forces to which the tissue is subjected, including those imposed by the surrounding tissues. In the case of a cylindrical vessel, these forces include the internal and external pressures. Forces due to compression or tethering associated with adjacent tissues can thus be accounted for in the external pressure.

### Data processing

Parameters of contractility diameter amplitude (*D*_amp_), contraction frequency (*f*), recovery time (*t*_recovery_), suction pressure amplitude (*Suction*_amp_), and peak pressure (*p*_max_), were quantified based on recorded pressure and diameter data using a custom algorithm in Matlab. The recovery time *t*_recovery_ is the time constant of an exponential curve fitted to the diameter versus time trace in diastole. Suction amplitude (*Suction*_amp_) is defined as the absolute value of the difference between inlet pressure and the minimum intralymphatic pressure. In the presence of a favourable axial pressure difference (*p*_in_ = 7, *p*_out_ = 3, *p*_e_ = 2 cmH_2_O), *Suction*_amp_ is more properly defined as the difference between end-diastolic pressure (lower than inlet pressure) and minimum intralymphatic pressure. Note that in the case of adverse axial pressure difference, end diastolic pressure and inlet pressure are the same, once refilling has been accomplished. We have established this definition primarily to facilitate a clear explanation of suction. A secondary goal is to relate our results to Guyton’s theory, in which such quantities were never precisely defined or measured. The parameters mentioned above are calculated for at least seven contractions. The average and standard deviation for each vessel are reported in Table 1.

### Experiments demonstrating transmission of suction

In order to investigate the degree to which suction is transmitted to upstream vessels, we performed two sets of experiments using two live vessels connected by a 115 µm diameter, 1 mm long connecting pipette (Fig. 5A). In the first set of experiments, a non-contracting mouse vessel was inserted upstream of the contracting rat vessel. Mouse iliac lymphatic vessels generally do not exhibit contractile behaviour. In the second set, a contracting lymphatic vessel was cut, and half of it placed in the upstream position. The physical separation by the interconnecting glass pipette was sufficient to prevent the vessels from contracting in phase with one another. This experiment enabled us to study suction under asynchronous contractions.

### Experiments demonstrating the effects of negative transmural pressure

Mouse inguinal axillary lymphatic vessels were dissected and cannulated for negative transmural pressure experiments with collagen surrounding the vessel. We studied the effect of negative transmural pressure by equal lowering of *p*_in_ and *p*_out_ from 3 cmH_2_O to 0 (with *p*_e_ = 0.5 cmH_2_O). External tethering was simulated by forming a collagen gel in the bath surrounding the vessel. The aim was to have an elastic medium that would adhere to the vessel wall, store elastic (potential) energy during vessel contraction, and then return that energy in the form of work applied to expand the vessel in diastole. The collagen gel contained 1.19 ml collagen I, 0.2 ml 10 × PBS, 0.027 g NaOH, and 0.58 ml dH_2_O. Following addition of collagen, the phasic contractions stopped briefly, but contractions resumed and stabilized after 10 min. The gel formation occurred in within the first 2–3 min, before regular contractions resumed. With collagen gelation around the vessel, a similar protocol of stepwise lowering of *p*_in_ = *p*_out_ was repeated when stabilized contractions resumed (Fig. 7).

### Mathematical model

A full explanation of the mechanisms behind suction requires knowledge of simultaneous flow rates and pressures in real time at several locations. For a number of reasons, *in situ* and *ex vivo* experiments are limited to providing only a subset of this information. For example, the isolated vessel experiments provide only pressure at certain locations. We and others have tried for years to employ particles and other techniques to perform flow measurements, but it is impossible to have a collection of particles that are perfectly neutrally buoyant. At the low fluid velocities inherent to these experiments, the particles either float or sink and clog the pipettes. We did manage to succeed in a short series of experiments with fluorescent microparticles, but the methodology is extremely cumbersome, interferes with pressure/diameter measurements, and is subject to its own uncertainties^46^. Most importantly, the images are only 2D, so one has to assume axisymmetry to estimate volume flow rate. Tracer dyes are prone to diffusion at the low Peclet numbers present in collecting lymphatics, and therefore unreliable as a quantitative flow measurement tool.

Given the long history of developing and validating our modelling techniques, we aimed to fill this gap using our lumped-parameter mathematical model of lymph flow to provide the required complete data set of flow and pressure^30,31^. Parameters for the model were based on the experimental measurements shown here. Briefly, equations of conservation of mass, conservation of momentum, and force balance on the vessel wall were solved for each lymphangion. Matlab’s routine ode15s was used to solve the system of nonlinear ordinary differential equations.

The valves were modelled using a sigmoidal function with minimum resistance to forward flow and maximum resistance to retrograde flow, with transition from closed to open states occurring at a slightly negative ∆*p* to represent bias of the valves to the open position^23,30^. The force balance on the vessel wall includes a term *f*_p_(*D*) which accounts for the non-linear passive behavior of the vessel, quantified via a curve fitted to experimental data obtained at the end of each experiment under Ca^2+^ free media. The force balance also incorporates active contractions via *f*_a_(*D*,*t*). The active tension generated by lymphatic muscle cells, *M*(*D*,*t*), includes a time-dependent component *M*_*t*_(*t*) as well as a diameter-dependent component *M*_d_(*D*). The current model improves upon previous versions of the model^30,47^, by implementing basal vessel tone estimated from experimental data. This is particularly important during the diastolic period, where this basal tone distinguishes the behaviour of the vessel from its completely passive state in Ca^2+^-free media. Parameters describing the diameter-dependent tension are chosen so that the resultant *M*_d_(*D*) recapitulates *ex vivo* contractile behaviour of the vessels used in these experiments at different levels of inlet and outlet pressures.

The waveform representing the time-dependence of contraction activation (*M*_*t*,_(*t*), Fig. 2C) is composed of two sinusoidal curves describing activation onset (*t* < 0.5 *t*_c1_, *M*_*t*,_ Fig. 2C, yellow) and slightly slower decay (0.5 *t*_c1_ < *t* < 0.5 (*t*_c1_ + *t*_c2_), Fig. 2C, purple), to create a contraction of fast onset and slower relaxation, as observed experimentally. Each contraction is followed by a diastolic period of full relaxation (*t*_r_, Fig. 2C, black). During the diastolic period *M*_*t*_ = *M*_b_, where *M*_b_ represents the basal tone.

The equations of the model are listed in the supplementary material, and parameters of the model are given in Table S1. Lymphangion and valve properties were assumed to be the same for all lymphangions. Also, based on the experiments reported here, all lymphangions were specified to contract synchronously, except for the simulation of two separate active vessels shown in Fig. 5F. Our previous work provides extensive exploration of the effects of asynchronous lymphangion contractions^30–32,48^.

A distinct advantage of the model is its ability to predict quantities that cannot be measured, such as the distribution of pressures and flow rates along the lymphangion chain. These quantities can then be used to calculate quantities of interest such as the work done by suction, based on the area of the pressure-diameter curve that falls below *p*_in_ (diastolic period only).

### Statistical Analyses

Matlab (R2016, MathWorks^®^) was used for all statistics. In Table 1, the average ± standard deviation for seven contractions of each vessel is reported for *N* = 5 vessels. For Fig. 3C, the average ± standard deviation of the fold changes in parameters of contractility (*D*_amp_, *f*, *t*_recovery_, *Suction*_amp,_ *p*_max_) with and without *R*_capillary_ for *N* = 5 vessels are shown. A *t*-test was performed to test the null hypothesis that the data come from a distribution with average of 1. For Fig. 4C, the average ± standard deviation for seven contractions of one vessel is reported. A *t*-test was performed to test the null hypothesis that the data collected at two measurement sites come from distributions with the same mean values. For Fig. 6, at each pressure scenario, the average ± standard deviation for seven contractions of each vessel is reported for *N* = 5 vessels. A *t*-test was performed to test the null hypothesis that the data come from a distribution with average of 0. For the plots demonstrating the ratio of *Suction*_amp_, a *t*-test was performed to test the null hypothesis that the data come from a distribution with average of 1. In all cases *p* < 0.01 was the criterion for significance.

## Electronic supplementary material

Supplementary Materials

## References

[1] Guyton AC, Coleman TG (1968). Regulation of interstitial fluid volume and pressure. Annals of the New York Academy of Sciences.

[2] Clough G, Smaje LH (1978). Simultaneous measurement of pressure in the interstitium and the terminal lymphatics of the cat mesentery. The Journal of Physiology.

[3] Granger HJ, Lane GA, Barnes GE, Lewis RE (1984). Dynamics and control of transmicrovascular fluid exchange. Edema.

[4] Hogan, R. D. The initial lymphatics and interstitial fluid pressure. In “Tissue Fluid Pressure and Composition” (A. R. Hargens, ed.). Williams and Wilkins, Baltimore., 155–163 (1981).

[5] Kvietys, P. The Gastrointestinal Circulation. *San Rafael* (*CA*): *Morgan & Claypool Life Sciences* (2010). 21452437

[6] Moriondo A, Mukenge S, Negrini D (2005). Transmural pressure in rat initial subpleural lymphatics during spontaneous or mechanical ventilation. American Journal of Physiology–Heart and Circulatory Physiology.

[7] Zweifach BW, Prather JW (1975). Micromanipulation of pressure in terminal lymphatics in the mesentery. American Journal of Physiology.

[8] Mislin, H. Structural and functional relations of the mesenteric lymph vessels. *Progress in Lymphology*. *Proceedings of the International Symposium on Lymphology*, 87–96 (1966).

[9] Schmid-Schönbein GW (1990). Microlymphatics and lymph flow. Physiological Reviews.

[10] Zawieja DC (2009). Contractile physiology of lymphatics. Lymphatic Research and Biology.

[11] Gashev AA, Zawieja DC (2001). Physiology of human lymphatic contractility: a historical perspective. Lymphology.

[12] Guyton AC, Granger HJ, Taylor AE (1971). Interstitial fluid pressure. Physiological Reviews.

[13] Hogan RD, Unthank JL (1986). The initial lymphatics as sensors of interstitial fluid volume. Microvascular Research.

[14] Negrini D, Moriondo A, Mukenge S (2004). Transmural pressure during cardiogenic oscillations in rodent diaphragmatic lymphatic vessels. Lymphatic Research and Biology.

[15] Gashev A, Orlov R, Zawieja D (2001). [Contractions of the lymphangion under low filling conditions and the absence of stretching stimuli. The possibility of the sucking effect]. Ross Fiziol Zh Im I M Sechenova.

[16] Trzewik J, Mallipattu SK, Artmann GM, Delano FA, Schmid-Schönbein GW (2001). Evidence for a second valve system in lymphatics: endothelial microvalves. The FASEB Journal.

[17] Parsons RJ, McMaster PD (1938). The effect of the pulse upon the formation and flow of lymph. The Journal of Experimental Medicine.

[18] McMaster PD (1947). The relative pressures within cutaneous lymphatic capillaries and the tissues. The Journal of Experimental Medicine.

[19] McMaster PD (1937). The lymphatics and lymph flow in the edematous skin of human beings with cardiac and renal disease. The Journal of Experimental Medicine.

[20] Benoit JN, Zawieja DC, Goodman AH, Granger HJ (1989). Characterization of intact mesenteric lymphatic pump and its responsiveness to acute edemagenic stress. American Journal of Physiology–Heart and Circulatory Physiology.

[21] Gashev AA, Davis MJ, Delp MD, Zawieja DC (2004). Regional variations of contractile activity in isolated rat lymphatics. Microcirculation.

[22] Davis MJ (2012). Intrinsic increase in lymphangion muscle contractility in response to elevated afterload. American Journal of Physiology–Heart and Circulatory Physiology.

[23] Davis MJ, Rahbar E, Gashev AA, Zawieja DC, Moore JE (2011). Determinants of valve gating in collecting lymphatic vessels from rat mesentery. American Journal of Physiology–Heart and Circulatory Physiology.

[24] Sloas D (2016). Estimation of the pressure drop required for lymph flow through initial lymphatic networks. Lymphat. Res. Biol..

[25] Bates DO, Levick JR, Mortimer PS (1994). Starling pressures in the human arm and their alteration in postmastectomy oedema. The Journal of Physiology.

[26] Grimaldi A (2006). Functional arrangement of rat diaphragmatic initial lymphatic network. American Journal of Physiology–Heart and Circulatory Physiology.

[27] Zamir M, Moore JE, Fujioka H, Gaver DP (2010). Biofluid mechanics of special organs and the issue of system control. Sixth International Bio-Fluid Mechanics Symposium and Workshop, March 28–30, 2008 Pasadena, California. Annals of Biomedical Engineering.

[28] Bridenbaugh EA (2013). An immunological fingerprint differentiates muscular lymphatics from arteries and veins. Lymphatic Research and Biology.

[29] Kunert C, Baish JW, Liao S, Padera TP, Munn LL (2015). Mechanobiological oscillators control lymph flow. Proceedings of the National Academy of Sciences.

[30] Jamalian S, Bertram CD, Richardson WJ, Moore JE (2013). Parameter sensitivity analysis of a lumped-parameter model of a chain of lymphangions in series. American Journal of Physiology–Heart and Circulatory Physiology.

[31] Jamalian S, Davis M, Zawieja D, Moore JE (2016). Network scale modeling of lymph transport and its effective pumping. Parameters..

[32] Bertram CD, Macaskill C, Moore JE (2011). Simulation of a chain of collapsible contracting lymphangions with progressive valve closure. Journal of Biomechanical Engineering.

[33] Preston NJ, Seers K, Mortimer PS (2004). Physical therapies for reducing and controlling lymphoedema of the limbs. Cochrane Database of Systematic Reviews.

[34] Rockson SG (2001). Lymphedema. The American Journal of Medicine.

[35] Wu TF, Carati CJ, MacNaughton WK, von der Weid P-Y (2006). Contractile activity of lymphatic vessels is altered in the TNBS model of guinea pig ileitis. American Journal of Physiology–Gastrointestinal and Liver Physiology.

[36] Cromer W (2015). Colonic insult impairs lymph flow, increases cellular content of the lymph, alters local lymphatic micro-environment and leads to sustained inflammation in the rat ileum. Inflammatory Bowel Diseases.

[37] Vasileiou AM (2011). Oedema in obesity; role of structural lymphatic abnormalities. International Journal of Obesity.

[38] Zawieja SD (2012). Impairments in the intrinsic contractility of mesenteric collecting lymphatics in a rat model of metabolic syndrome. American Journal of Physiology–Heart and Circulatory Physiology.

[39] Blum KS (2014). Chronic high-fat diet impairs collecting lymphatic vessel function in mice. PLoS One.

[40] Akl TJ, Nagai T, Coté GL, Gashev AA (2011). Mesenteric lymph flow in adult and aged rats. American Journal of Physiology–Heart and Circulatory Physiology.

[41] García-Cruz D (2011). Cantu syndrome and lymphoedema. Clinical Dysmorphology.

[42] Davis MJ, Zawieja DC, Gashev AA (2006). Automated measurement of diameter and contraction waves of cannulated lymphatic microvessels. Lymphatic Research and Biology.

[43] Davis MJ, Davidson J (2002). Force-velocity relationship of myogenically active arterioles. American Journal of Physiology–Heart and Circulatory Physiology.

[44] Bertram CD, Macaskill C, Moore JE (2011). Simulation of a chain of collapsible contracting lymphangions with progressive valve closure. ASME Journal of Biomechanical Engineering.

[45] Akl, T. J., Nepiyushchikh, Z. V., Gashev, A. A., Zawieja, D. C. & Coté, G. L. Measuring contraction propagation and localizing pacemaker cells using high speed video microscopy. *Journal of Biomedical Optics* **16**(2), 026016-1–026016-9, doi:10.1117/1.3544512 (2011). 10.1117/1.3544512 PMC3065345 21361700

[46] Margaris KN, Nepiyushchikh Z, Zawieja DC, Moore JJ, Black RA (2016). Microparticle image velocimetry approach to flow measurements in isolated contracting lymphatic vessels. Journal of Biomedical Optics.

[47] Bertram CD, Macaskill C, Davis MJ, Moore JE (2014). Development of a model of a multi-lymphangion lymphatic vessel incorporating realistic and measured parameter values. Biomechanics and Modeling in Mechanobiology.

[48] Bertram CD, Macaskill C, Davis MJ, Moore JE (2016). Consequences of intravascular lymphatic valve properties: a study of contraction timing in a multi-lymphangion model. American Journal of Physiology–Heart and Circulatory Physiology.

