# Supplementary material for: Demonstration and Analysis of the Suction Effect for Pumping Lymph from Tissue Beds at Subatmospheric Pressure
Source: Sci Rep. 2017 Sep 21;7:12080. doi: 10.1038/s41598-017-11599-x (PMC5608746; doi:10.1038/s41598-017-11599-x)
Supplement: Supplementary file 1 — Supplementary Materials [file 41598_2017_11599_MOESM1_ESM.pdf]

Supplementary Materials: Information on Mathematical Model including Equations and Parameter Values

The equations of the model are listed below:

Conservation of mass:

$$Q_{i+1} = Q_i - \frac{\pi D_i L_i}{2}\frac{dD_i}{dt} \ .$$

Conservation of momentum:

$$p_{\text{in}} - p_a = R_{\text{pipette,up}}\, Q_1; \quad p_b - p_{\text{out}} = R_{\text{pipette,down}}\, Q_{i+1};$$

$$p_{i-1,2} - p_{i1} = R_{\text{V}i}\, Q_i\,; \quad \frac{p_{i1} - p_{im}}{L_i} = \frac{64\mu Q_i}{\pi D_i^4}\,;$$

$$\frac{p_{im} - p_{i2}}{L_i} = \frac{64\mu Q_{i+1}}{\pi D_i^4}\,; \quad p_{i2} - p_{i+1,1} = R_{\text{V}i+1}\, Q_{i+1}\,,$$

where $R_{\text{V}i} = R_{\text{V}i}\left(p_{i-1,2} - p_{i1}\right)$ and $R_{\text{V}i+1} = R_{\text{V}i+1}\left(p_{i2} - p_{i+1,1}\right)$.

Conservation of momentum:

$$p_{\text{in}} - p_a = R_{\text{capillary}}\, Q_1; \quad p_{\text{b}} - p_{\text{out}} = R_{\text{b}}\, Q_{i+1};$$

and $R_{\text{V}i} = R_{\text{Vn}} + R_{\text{Vx}}\left(\dfrac{1}{1+e^{-s_{\text{f}}(\Delta p - \Delta p_{\text{f}})}} + \dfrac{1}{1+e^{s_{\text{o}}(\Delta p - \Delta p_{\text{o}})}} - 1\right)$

Vessel wall force balance:

$$\Delta p_{tm} = p_{im} - p_e = f_p(D) + f_a(D,t) \ ;$$

Passive behavior:

$$f_p(D) = c_1 \exp(c_2 D_i) + c_3 \exp(c_4 D_i) + c_5 D_i + c_6 + \frac{c_7}{(D_i)^3}\,,$$

with curve fit parameters $c_1 - c_7$ as listed below:

$$c_1 = 6.01 \times 10^{-5} \frac{dyn}{cm^2}$$

$$c_2 = 8.96 \times 10^{2} \frac{1}{cm}$$

$$c_3 = 1.23 \times 10^{-18} \frac{dyn}{cm^2}$$

$$c_4 = 2.70 \times 10^{3} \frac{1}{cm}$$

$$c_5 = -1.94 \times 10^{4} \frac{dyn}{cm^3}$$

$$c_6 = 5.56 \times 10^{2} \frac{dyn}{cm^2}$$

$$c_7 = -2 \times 10^{-3} dyn.cm$$

Active component:

$f_{\mathrm{a}}(D,t) = \dfrac{2M(D,t)}{D_i}$ and $M(D,t) = M_t(t) \times M_d(D)$.

Time-dependent tension:

$t < 0.5\ t_c,\ M_t(t) = M_a(1-\cos(2t/t_c))/2 + M_b$

$0.5\ t_c < t < 0.5\ (t_c + t_r),\ M_t(t) = M_a(1-\cos(2(t+0.5(t_r - t_c))/t_r))/2 + M_b$

$t > 0.5\ (t_c + t_r),\ M_t(t) = M_b$

where $t \leq 0.5\ t_{\mathrm{c}}$ defines the activation onset period, $0.5\ t_{\mathrm{c}} \leq t \leq 0.5\ (t_{\mathrm{c}} + t_{\mathrm{r}})$ defines the relaxation period; and the following contraction begins at $0.5\ (t_{\mathrm{c}} + t_{\mathrm{r}}) + t_{\mathrm{d}}$.

Diameter-dependent tension

$$M_{\mathrm{d0}}(D) = \frac{5.5}{1+e^{-s_{\mathrm{d0}}(D_i - D_{\mathrm{d0}})}},\ s_{d0} = 2000\frac{1}{cm},\ D_{d0} = 0.019cm$$

$$M_{\mathrm{d1}}(D) = \frac{0.5}{1+e^{-s_{\mathrm{d1}}(D_i - D_{\mathrm{d1}})}},\ s_{d1} = 250\frac{1}{cm},\ D_{d1} = 0.021cm$$

$$M_{\mathrm{d2}}(D) = \frac{2}{1+e^{s_{\mathrm{d2}}(D_i - D_{\mathrm{d2}})}},\ s_{\mathrm{d2}} = 1000\frac{1}{cm},\ D_{\mathrm{d2}} = 0.05cm$$

$$M_{\mathrm{d}}(D) = (M_{\mathrm{d0}}(D) + M_{\mathrm{d1}}(D) + M_{\mathrm{d2}}(D) - 2)/6$$

Description	Parameter	Value	Units
Valve parameters			
Valve failure pressure	Δp_f	–18.4	cmH_2O
Valve failure slope	s_f	0.049	cm^2/dyn
Minimum valve resistance	R_{Vn}	8×10^6	$g/(cm^4\ s)$
Maximum valve resistance	R_{Vx}	9.9×10^9	$g/(cm^4\ s)$
Valve opening pressure	Δp_o	–15	dyn/cm^2
Valve opening slope	s_o	0.4	cm^2/dyn
Non-valve parameters			
Fluid viscosity	μ	0.009	g/(cm s)
Lymphangion length	L	0.1	cm
Number of lymphangions per vessel	n_v	4	-
Contraction period	t_c	2	s
Relaxation period	t_r	3	s
Diastolic period	t_d	7.5	s
Inter-lymphangion time difference	(via Δt)	0	s
Vessel tone	M_b	500	dyn/cm
Active tension	M_a	6000	dyn/cm
External pressure	p_e	0.5	cmH_2O
Resistance of the upstream pipette	$R_{pipette,up}$	1.84×10^7	$g/cm^4\ s$
Resistance of the downstream pipette	$R_{pipette,down}$	7.2×10^6	$g/cm^4\ s$
Resistance of the capillary tubing	$R_{capillary}$	4.8×10^7	$g/cm^4\ s$

Table S1. Parameters used in the numerical model, including their definition and baseline values.
